# 808. Outcomes of Invasive Candidiasis Stratified by Antifungal Therapy

**DOI:** 10.1093/ofid/ofad500.853

**Published:** 2023-11-27

**Authors:** Maggie Box, Lara Anwar, Kyle Molina

**Affiliations:** Scripps Memorial Hospital La Jolla, La Jolla, California; Scripps Memorial Hospital La Jolla, La Jolla, California; Scripps Health, San Diego, California

## Abstract

**Background:**

IDSA guidelines for invasive candidiasis recommend echinocandins (ECH) as first-line agents for initial therapy, with fluconazole (FLU) as an acceptable alternative. Transitioning from an ECH to FLU is also recommended in clinically stable patients with FLU susceptible isolates. All-cause mortality is high for candidemia, typically due to medical comorbidities in patients who develop candidiasis. The objective of this study is to report outcomes of invasive candidiasis stratified by antifungal therapy received, comparing micafungin (MIC), fluconazole, and micafungin to fluconazole step down therapy.

**Methods:**

This multicenter, retrospective study was conducted at Scripps Health from 6/1/2019 – 6/30/2022. Adult patients with culture confirmed invasive candidiasis were included. Patients were excluded if they were pregnant, care was withdrawn within 7 days of treatment initiation, or had prolonged course of therapy due to uncontrolled source. The primary endpoint was 28-day mortality based on antifungal therapy. Secondary outcomes included time-to-death among and duration of therapy trends.

**Results:**

A total of 95 patients were included. *Candida glabrata* was most frequently isolated (43.2%) followed by *Candida albicans* (36.8%). There was no difference in 28-day mortality among patients receiving fluconazole only (15.4%, n = 13), micafungin only (20.5%, n = 44), micafungin only due to fluconazole resistance (12.5%, n = 8), and micafungin to fluconazole step down therapy (13.3%, n = 30), p = 0.849. Among patients who died, there was no difference in time-to-death between groups (p = 0.678) or mortality by organism (*C. albicans* 37.5%, p = 0.952 and *C. glabrata* 43.8%, p = 0.958). Six patients with non-*C. albicans* isolates that were susceptible dose-dependent (SDD) to FLU were successfully treated with FLU step-down therapy with a dose/MIC ratio of > 100.

**Conclusion:**

There was no difference in 28-day mortality or time-to-death between patients who received fluconazole versus micafungin for invasive candidiasis. Fluconazole step down therapy (including oral) is a viable option for patients who are candidates, including non-*C. albicans* SDD organisms.

**Disclosures:**

**All Authors**: No reported disclosures

